# Electronic Structure of Graphene-Doped PEDOT:PSS and Its Influence on Energy-Level Alignment with p-Type Organic Semiconductor ZnPc

**DOI:** 10.3390/ma19020295

**Published:** 2026-01-12

**Authors:** Woojin Shin, Hyunbok Lee

**Affiliations:** Department of Semiconductor Physics, Institute of Forest Science, Kangwon National University, 1 Gangwondaehak-gil, Chuncheon-si 24341, Gangwon-do, Republic of Korea; swj452@gmail.com

**Keywords:** PEDOT:PSS, graphene, UPS, XPS, energy-level alignment

## Abstract

Poly (3,4-ethylenedioxythiophene polystyrene sulfonate) (PEDOT:PSS) is a solution-processable hole transport layer known for its high work function and excellent hole mobility. The incorporation of graphene serves as an effective strategy to augment the hole-transport properties of PEDOT:PSS. In this study, the electronic structure of graphene-doped PEDOT:PSS (G-PEDOT:PSS) was investigated using X-ray photoelectron spectroscopy (XPS) and ultraviolet photoelectron spectroscopy (UPS). It was found that the work function of PEDOT:PSS increases with graphene doping concentration, rising from 4.86 eV for undoped PEDOT:PSS to 5.03 eV for PEDOT:PSS incorporating 10 wt% graphene. The impact of this modification on the energy-level alignment with zinc phthalocyanine (ZnPc), which is a prototypical p-type organic semiconductor, was examined through in situ XPS and UPS analyses. Despite the increased work function, the hole injection barriers for both PEDOT:PSS and G-PEDOT:PSS to ZnPc were determined to be identical at 0.26 eV. This lack of change in the barrier is explicitly attributed to Fermi-level pinning, where the integer charge transfer level of ZnPc is pinned to the Fermi level of the substrate, preventing a further reduction in the energy offset. That said, for other p-type organic semiconductors with higher ionization energies, the use of G-PEDOT:PSS could potentially enable more efficient hole injection.

## 1. Introduction

Poly (3,4-ethylenedioxythiophene polystyrene sulfonate) (PEDOT:PSS) is a polymeric material that is widely employed in electronic devices such as light-emitting diodes, solar cells, sensors, and thermoelectric generators [1,2,3,4,5,6]. Owing to its high work function, excellent hole mobility, and solution processability, PEDOT:PSS effectively serves as both the electrode and the hole transport layer (HTL). Structurally, it exhibits a core–shell configuration in which the conductive PEDOT domains form the core and the insulating PSS acts as the shell. This morphology enables tunable electrical conductivity, ranging from semiconducting HTL behavior at low PEDOT contents to highly conductive electrode performance at higher PEDOT-to-PSS ratios [7].

To achieve high-efficiency organic electronic devices, it is necessary to overcome the challenges posed by the limited hole transport ability of PEDOT:PSS. Various doping strategies have been explored to enhance the electrical and interfacial properties of PEDOT:PSS materials [8], with graphene and its chemically modified derivatives used as dopants demonstrating particularly strong effects [9]. Graphene doping increases the work function, electrical conductivity, surface wettability, and environmental stability of PEDOT:PSS, thereby improving the overall performance of optoelectronic devices [10,11,12]. However, the fundamental changes in the electronic structure of PEDOT:PSS induced by different graphene concentrations remain poorly understood, hindering rational optimization of device architectures.

Graphene dispersion in water (GDW) which is a surfactant-free suspension of partially oxidized graphene flakes offers excellent compatibility with aqueous PEDOT:PSS processing. GDW can function both as a conductive dopant and as an electrode material [13]. Despite its promising properties, the electronic structure of GDW-doped PEDOT:PSS (G-PEDOT:PSS) has not been characterized in detail to date, limiting its broader application potential.

In organic electronic devices, charge-carrier injection from electrodes plays a key role because organic semiconductors lack intrinsic free carriers. The injection efficiency is governed by the energy barrier between the electrode’s Fermi level and the frontier molecular orbitals of the organic semiconductor, namely, the highest occupied molecular orbital (HOMO) for hole injection and the lowest unoccupied molecular orbital (LUMO) for electron injection. Increasing the electrode work function can facilitate hole injection; thus, graphene doping in PEDOT:PSS offers a potential route for reducing the hole injection barrier by modifying its electronic structure.

However, the interfacial energy-level alignment in organic heterojunctions often deviates from the Schottky–Mott limit (i.e., vacuum-level alignment) owing to the formation of interface dipoles and Fermi-level pinning effects [14,15,16], which can inhibit the reduction in the injection barrier even when the electrode work function is increased. Therefore, direct characterization of the actual interfaces is essential for the elucidation of the underlying charge injection mechanisms.

In this study, we systematically investigate the electronic structure of G-PEDOT:PSS at different graphene doping concentrations. X-ray photoelectron spectroscopy (XPS) and ultraviolet photoelectron spectroscopy (UPS) were employed to examine the variations in the core and valence levels. Furthermore, in situ measurements were performed to analyze the alignment of the energy levels at the interfaces between pristine PEDOT:PSS or G-PEDOT:PSS and zinc phthalocyanine (ZnPc). ZnPc was selected as a prototypical p-type organic semiconductor for this study, as it is widely employed as a key active material in organic solar cells and thin-film transistors. Beyond its technological relevance, ZnPc serves as an effective probe to investigate energy-level alignment due to its well-characterized electronic structure and an ionization energy (IE) of approximately 5 eV. This IE value makes ZnPc particularly suitable for demonstrating whether an increase in the electrode work function translates to a reduced hole injection barrier or is constrained by Fermi-level pinning according to the integer charge transfer (ICT) model. These analyses provide direct comparisons of the hole-injection barriers and offer valuable insights into the optimization of PEDOT:PSS-based HTLs for advanced organic electronic devices.

## 2. Materials and Methods

PEDOT:PSS (Clevios^TM^ P VP AI 4083, concentration: 1.5 wt%; Heraeus Epurio GmbH, Leverkusen, Germany) and GDW (purity: 99.5%, concentration: 1.0 wt%; Nanografi Nano Technology Co., Ankara, Turkey) were used as received. To prepare the G-PEDOT:PSS dispersion, GDW was added to the PEDOT:PSS solution at the specified weight percentages of 0, 2, 5, 10, and 20 wt%. The resulting mixtures were ultrasonicated to ensure homogeneous dispersion and to prevent graphene aggregation prior to film deposition.

PEDOT:PSS and G-PEDOT:PSS films were deposited onto indium tin oxide (ITO)-coated glass substrates by spin-coating first at 500 rpm for 5 s and then at 3000 rpm for 60 s. To ensure the removal of residual solvents and to promote a more uniform film morphology, a thermal treatment was performed at 150 °C for 10 min.

Surface morphology was examined using atomic force microscopy (AFM) in the tapping mode with an FX40 microscope (Park Systems Co., Suwon, Republic of Korea). The electronic structures were analyzed by XPS and UPS using a PHOIBOS 150 analyzer (SPECS Surface Nano Analysis GmbH, Berlin, Germany) equipped with an Al K_α_ (hν = 1486.7 eV) X-ray source and a He I_α_ (hν = 21.22 eV) discharge lamp for XPS and UPS measurements, respectively. The base pressure of the analysis chamber was 4 × 10^−10^ Torr. A detailed description of the in situ analysis system can be found elsewhere [17].

ZnPc (purity: 99%; Jilin OLED Material Tech Co. Ltd., Changchun, China) was thoroughly degassed prior to deposition and thermally evaporated in stepwise manner at a rate of 0.01 nm s^−1^. The base pressure of the deposition chamber was 8 × 10^−8^ Torr. To ensure accurate measurements, the deposition rate and total thickness were monitored using a quartz crystal microbalance, with calibration verified by cross-sectional scanning electron microscope. To accurately determine the secondary electron cutoff (SECO), a sample bias of −5 V was applied during the UPS measurements.

## 3. Results and Discussion

**Figure 1:**
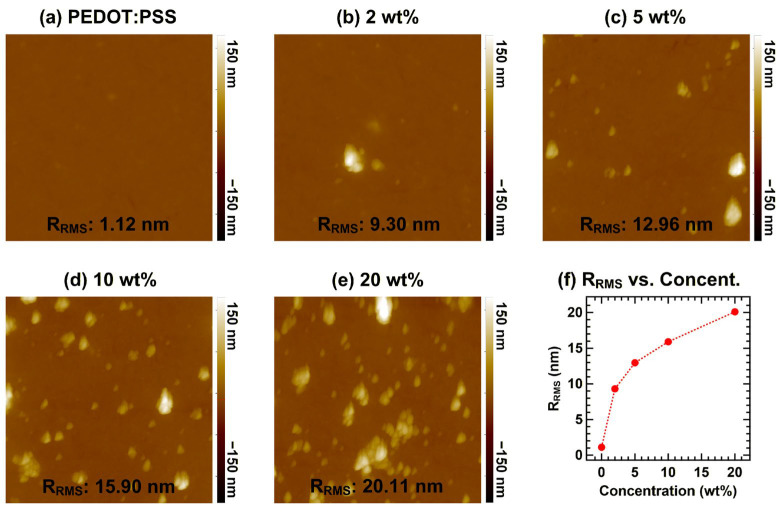
AFM images of PEDOT:PSS films with different graphene doping concentrations: (**a**) 0, (**b**) 2, (**c**) 5, (**d**) 10, and (**e**) 20 wt%. (**f**) Variation in R_RMS_ as a function of graphene doping concentration. The scan area for each image is 5 × 5 μm^2^, and a uniform color scale (height range: −150 to 150 nm) is applied to all images to allow direct comparison.

First, we investigated the morphological changes in the PEDOT:PSS films induced by graphene doping. Figure 1 shows the AFM images of graphene-doped PEDOT:PSS with various doping concentrations. The pristine PEDOT:PSS surface exhibited a very smooth morphology characterized by a uniform topography. With increasing graphene content, the film surface became progressively rougher due to the incorporation of graphene flakes, which appeared as bright protrusions in the AFM images. The root-mean-square roughness (R_RMS_) increased markedly from 1.12 nm for pristine PEDOT:PSS to 9.30, 12.96, 15.90, and 20.11 nm for graphene doping levels of 2, 5, 10, and 20 wt%, respectively. To better visualize this morphological transition, the variation in R_RMS_ as a function of doping concentration is plotted in Figure 1f.

The substantial roughening observed at higher graphene concentrations indicates that excessive incorporation of graphene flakes disrupts the surface uniformity of the film. Such morphological changes can adversely affect the formation of interfaces with overlying organic semiconductors, potentially inducing leakage currents or poor contact quality. Notably, the electronic benefit—specifically the increase in work function—plateaus at 10 wt% (5.03 eV, see the UPS results), implying that further doping beyond this concentration degrades the morphology without offering additional gains in energy-level tuning. Consequently, the effectiveness of graphene doping in tuning the PEDOT:PSS work function is constrained by the simultaneous increase in surface roughness. A strategic balance must be maintained to maximize electronic gains while minimizing morphological impacts, which is critical for achieving optimal efficiency in optoelectronic architectures.

**Figure 2:**
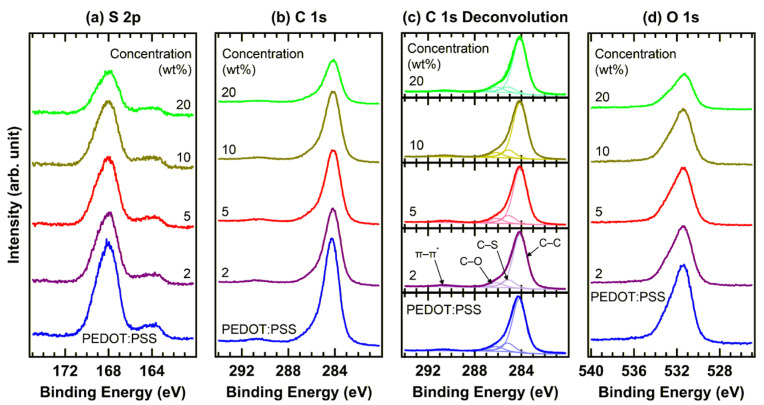
XPS spectra of the (**a**) S 2p, (**b**) C 1s, (**c**) deconvoluted C 1s, and (**d**) O 1s regions of PEDOT:PSS films with different graphene doping concentrations (0, 2, 5, 10, and 20 wt%). The π–π* satellite is a shake-up peak due to the loss of photoelectron kinetic energy caused by the excitation of the π (HOMO) to π* (LUMO) transition.

**Figure 3:**
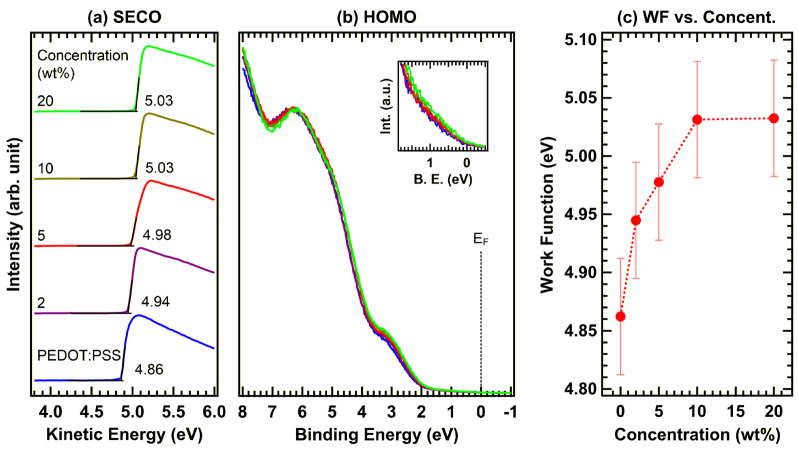
UPS spectra of the (**a**) SECO and (**b**) HOMO regions of PEDOT:PSS films with different graphene doping concentrations [0 (blue), 2 (purple), 5 (red), 10 (olive), and 20 (green) wt%]. (**c**) Variation in the work function as a function of graphene concentration. The error bars are estimated to be within ±0.05 eV based on the instrumental resolution and experimental reproducibility.

Figure 2 shows the XPS spectra of G-PEDOT:PSS films with various doping concentrations. In the S 2p region (Figure 2a), two distinct spectral features are observed: the lower-binding-energy component (161.9–165.6 eV) corresponds to the conducting PEDOT moieties while the higher-binding-energy component (165.9–171.2 eV) is assigned to the insulating PSS moieties [7,18,19,20,21,22]. The ratio of PEDOT to PSS reflects the relative surface exposure of the conducting phase, with a higher ratio generally associated with improved film conductivity [23,24,25]. No noticeable changes in the PEDOT/PSS ratio were detected upon graphene doping; rather, a gradual decrease in the overall signal intensity was observed. This attenuation is attributed to the partial coverage of the PEDOT:PSS surface by graphene flakes, which is consistent with the AFM results shown in Figure 1. Upon the incorporation of graphene, the S 2p peaks exhibited a shift of ~0.1 eV toward lower binding energies, aligning with the shifts recorded in the UPS spectra (see Figure 3).

An in-depth examination of the C 1s spectra provides further insight into the electronic structural modifications within G-PEDOT:PSS [26,27,28]. The C 1s region and its corresponding deconvolution (Figure 2b,c), and Table 1 exhibit characteristic peaks at 284.2, 285.2, 286.2, and 290.7 eV, assigned to C–C, C–S, C–O, and π–π* satellites, respectively. The satellite feature at higher binding energy originates from energy-loss processes related to the excitation of HOMO electrons to the LUMO. Consistent with the trend seen in the S 2p spectra, the C 1s core levels shifted toward lower binding energies upon graphene incorporation. Meanwhile, the relative intensity of the C–S peak from PEDOT:PSS matrix gradually diminishes with increasing graphene content, suggesting an increased surface concentration of graphene-related species. Because the C–C and C–O binding energies of PEDOT:PSS and the GDW dopant are inherently similar, no new peaks emerged despite the increased loading. This spectral stability confirms the absence of major chemical environment alterations.

For the O 1s region (Figure 2d), the dominant peak located at 531.4 eV is attributed to O–S bonds, while a secondary feature at a higher binding energy (533.1 eV) corresponds to O–C bonds. Consistent with other core levels, the O 1s peak shifted toward lower binding energies by ~0.1 eV. With increasing graphene doping, the overall spectral shape remains largely unchanged even though the peak intensities decrease. This attenuation is attributed to the increasing contribution of the carbon-rich GDW component at the PEDOT:PSS surface, which partially suppresses the oxygen-related signals.

Overall, the XPS results indicate that graphene was well-integrated into the PEDOT:PSS matrix without inducing significant chemical shifts, suggesting that the interactions between graphene and PEDOT:PSS are primarily physical rather than chemical. However, the incorporation of graphene altered the surface composition and electronic environment of the film.

Figure 3 shows the UPS spectra of G-PEDOT:PSS films with different graphene doping concentrations. Plots of the UPS intensity as a function of kinetic energy in the SECO regions (Figure 3a) allowed direct determination of the work function from the cutoff position. These spectra were normalized to highlight the relative shifts. The pristine PEDOT:PSS exhibited a work function of 4.86 eV. With increasing graphene concentration, the work function progressively rose to 4.94, 4.98, and 5.03 eV for 2, 5, and 10 wt% doping, respectively. The experimental uncertainty for the UPS measurements was estimated to be ±0.05 eV, derived from the Au Fermi-edge broadening and experimental reproducibility. This ensures that the observed work function increase of 0.17 eV is physically meaningful, as it significantly exceeds the instrumental resolution (Figure 3c).

However, a further increase in the concentration to 20 wt% produced no additional increase in the work function. This behavior is governed by the intrinsic electronic properties of the dopant. GDW, which contains graphene oxide, possesses intrinsically high work functions [13,29,30,31], and is present on the PEDOT:PSS surface (Figure 1). Specifically, the work function of the composite at this concentration reaches 5.03 eV, which aligns with the intrinsic work function of pristine GDW (5.03 eV) as recently reported [32]. This suggests that at 10 wt%, the surface of the film is energetically dominated by GDW components. Because the composite’s work function is theoretically capped by the work function of the pure dopant phase, further increases in GDW concentration do not result in a higher work function value, as the system has reached its maximum potential defined by the dopant’s electronic structure.

The HOMO region spectra (Figure 3b) were normalized to the PSS peak at approximately 6.3 eV, which served as a stable reference. Across all doping concentrations, the spectral envelopes remained nearly identical, suggesting that the valence band structure of PEDOT:PSS is largely preserved upon graphene addition. The similarity in electronic structure is attributed to the presence of hybridized π-states at approximately 3 eV and O 2p-related states in both materials [7,13,33,34]. Notably, a rigid shift of ~0.2 eV toward lower binding energies was observed, consistent with the shifts identified in the SECO region. Furthermore, the inset shows an increase in the density of states near the Fermi level with increasing graphene concentration. This feature suggests enhanced electrical conductivity, consistent with improved charge transport characteristics which are advantageous for optoelectronic devices that require efficient hole injection and transport.

**Figure 4:**
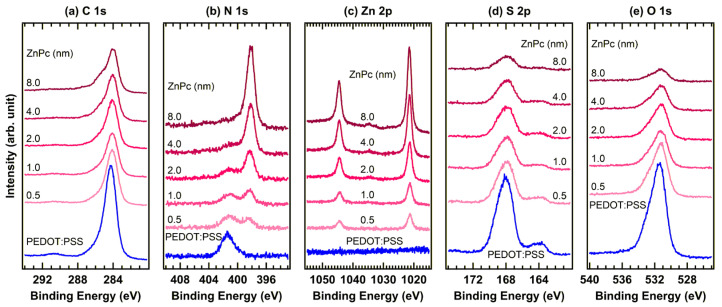
In situ XPS spectra of the (**a**) C 1s, (**b**) N 1s, (**c**) Zn 2p, (**d**) S 2p, and (**e**) O 1s regions of the PEDOT:PSS/ZnPc interface.

Figure 4 shows in situ XPS spectra of ZnPc deposited stepwise onto pristine PEDOT:PSS. In the C 1s region (Figure 4a), in addition to the characteristic PEDOT:PSS peaks shown in Figure 2, new features appeared upon ZnPc deposition. These peaks are located at 284.0, 285.4, and 287.4 eV and correspond to C–C and C–N bonds, and a satellite feature, respectively [35]. With increasing ZnPc coverage, the intensities of the PEDOT:PSS-related C 1s signals diminished, confirming the progressive overlayer formation.

In the N 1s region (Figure 4b), a weak peak at 401.4 eV was observed for pristine PEDOT:PSS, likely arising from trace nitrogen-containing residues remaining from polymer synthesis [36,37]. Upon ZnPc deposition, a dominant N 1s peak emerged at 398.1 eV, characteristic of C–N bonds within the ZnPc macrocycle, confirming the successful deposition of the organic semiconductor.

The Zn 2p region (Figure 4c) shows the appearance of Zn 2p_3/2_ and 2p_1/2_ peaks at 1021.4 and 1044.5 eV, respectively, arising from spin–orbit splitting. Their increasing intensities with increasing deposition thickness indicate the controlled stepwise growth of ZnPc on the PEDOT:PSS surface.

In the S 2p region (Figure 4d), the relative intensity ratio between the PEDOT and PSS components remained unchanged after ZnPc deposition, suggesting that the underlying PEDOT:PSS network was structurally preserved. Correspondingly, the S 2p and O 1s signals in (Figure 4e) were gradually attenuated as the ZnPc layer thickened, which agrees well with the uniform surface coverage by the overlayer. Notably, no significant binding-energy shifts were detected in any of the core-level spectra, indicating the absence of strong chemical interactions and charge transfer at the interface.

**Figure 5:**
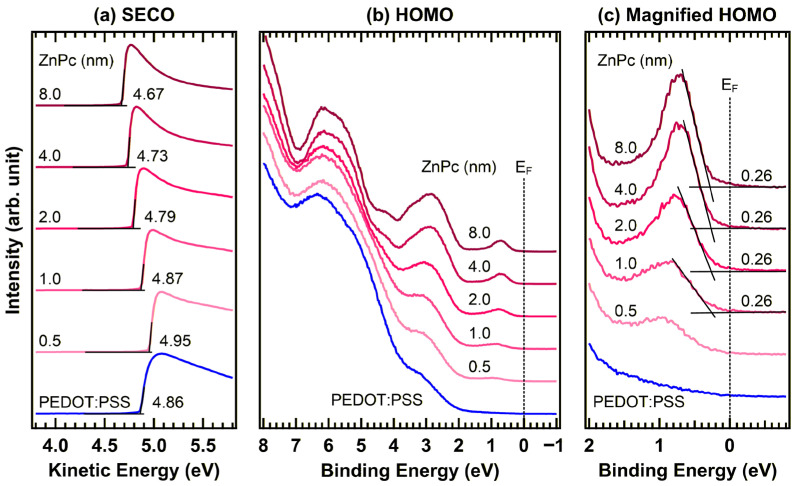
In situ UPS spectra of the (**a**) SECO and (**b**) HOMO regions, and (**c**) a magnified view of the HOMO region of the PEDOT:PSS/ZnPc interface.

Figure 5 shows the in situ UPS spectra of ZnPc deposited on pristine PEDOT:PSS. In the SECO region Figure 5a, the pristine PEDOT:PSS film exhibited a work function of 4.86 eV. Upon deposition of a 0.5 nm thick ZnPc layer, the work function increased slightly to 4.95 eV but subsequently decreased with further ZnPc growth, reaching 4.87, 4.79, 4.73, and 4.67 eV at the thicknesses of 1.0, 2.0, 4.0, and 8.0 nm, respectively. The initial opposite SECO shift at ultrathin coverage may arise from a thickness-dependent evolution of the interface dipole, driven by changes in molecular orientation [35,38,39,40]. Specifically, at the sub-monolayer stage, the orientation of ZnPc molecules (potentially governed by strong substrate–adsorbate interactions) could induce a transient surface dipole that increases the work function. As the film thickness increases, a gradual reorientation driven by dominant intermolecular interactions likely alters the dipole contribution, resulting in the subsequent decrease in work function. While a definite analysis of these structural transitions is beyond the scope of this work, these observations highlight the sensitivity of energy-level alignment to early-stage interface formation. Although the SECO shifts did not fully saturate at 8.0 nm, further analysis was not conducted because thicker films exhibited substantial peak shifts, raising concerns regarding charging effects [41,42]. However, the available data are sufficient to describe the evolution of the interfacial electronic states.

In the HOMO region (Figure 5b), progressive ZnPc deposition gives rise to characteristic valence features at 0.7, 2.8, 3.5, 5.6, and 6.2 eV, with their intensities increasing with film thickness, confirming sequential and uniform layer growth. A magnified view of the HOMO is shown in Figure 5c. The first discernible HOMO onset, observed at 0.26 eV for the 1.0 nm thick ZnPc layer (approximately corresponding to monolayer completion), remains unchanged with further film growth. This invariance indicated the absence of significant energy-level bending within the ZnPc layer. These results are consistent with the XPS findings shown in Figure 4 which also reveal no core-level binding-energy shifts.

**Figure 6:**
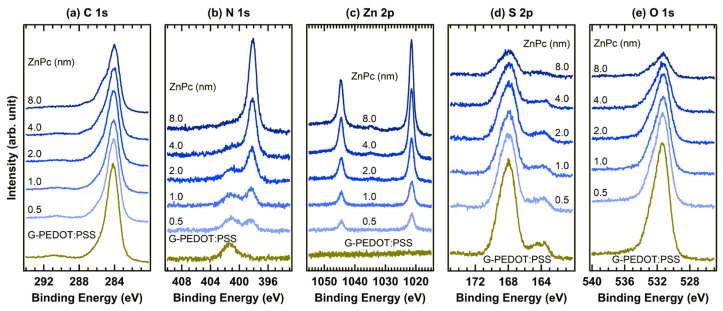
In situ XPS spectra of the (**a**) C 1s, (**b**) N 1s, (**c**) Zn 2p, (**d**) S 2p, and (**e**) O 1s regions of the G-PEDOT:PSS (10 wt%)/ZnPc interface.

To examine the influence of graphene doping on the interfacial electronic properties, the in situ XPS spectra of ZnPc deposited on G-PEDOT:PSS were measured, as shown in Figure 6. Because the increase in the work function saturated at a graphene doping level of 10 wt%, G-PEDOT:PSS with a graphene content of 10 wt% was used. In the C 1s region (Figure 6a), the dominant C–C peak is located at 284.1 eV, accompanied by C–O and satellite features at higher binding energies, which is consistent with the G-PEDOT:PSS spectra presented in Figure 2b. Upon ZnPc deposition, new peak characteristics of ZnPc appeared at binding energies similar to those observed for the PEDOT:PSS/ZnPc interface (Figure 4a), indicating a comparable interfacial composition.

In the N 1s spectrum (Figure 6b), the spectrum gradually transitioned from a weak PEDOT:PSS-related component to a dominant ZnPc peak at 398.1 eV, corresponding to C–N bonding within the phthalocyanine ring. Likewise, the Zn 2p_3/2_ and 2p_1/2_ peaks observed at 1021.4 and 1044.5 eV, respectively, (Figure 6c) increased steadily with increasing film thickness, indicating controlled ZnPc layer growth. These binding energies closely match those at the PEDOT:PSS/ZnPc interface, suggesting that graphene doping does not significantly alter the chemical environment of the Zn and N atoms in the overlayer.

In the S 2p and O 1s regions (Figure 6d,e), the peak positions and shapes remained essentially unchanged during ZnPc deposition, whereas their intensities decreased gradually with increasing ZnPc thickness. This attenuation reflects the uniform ZnPc coverage and indicates that the underlying G-PEDOT:PSS layer remains chemically stable. The absence of binding-energy shifts across all core-level spectra confirms a weak interfacial interaction and the lack of a chemical reaction between ZnPc and G-PEDOT:PSS, which is consistent with the formation of a predominantly physical interface.

**Figure 7:**
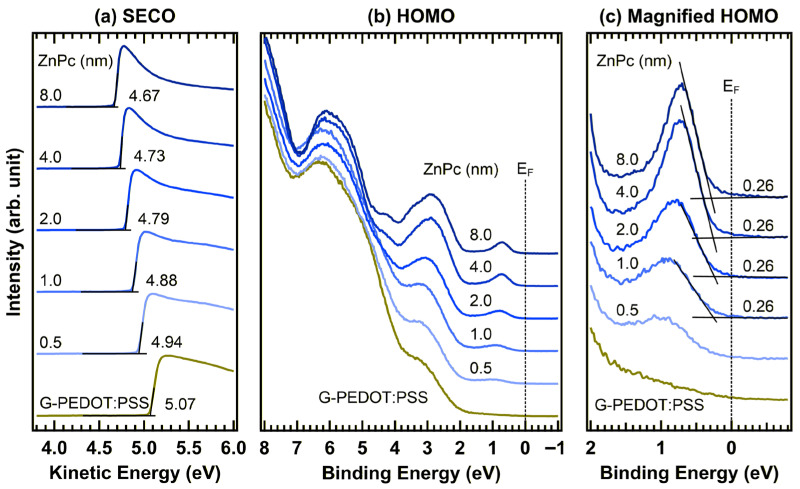
In situ UPS spectra of the (**a**) SECO and (**b**) HOMO regions, and (**c**) a magnified view of the HOMO region of the G-PEDOT:PSS (10 wt%)/ZnPc interface.

Figure 7 shows the in situ UPS spectra of ZnPc deposited on G-PEDOT:PSS. As shown in Figure 7a, the work function of G-PEDOT:PSS was determined to be 5.07 eV, in agreement with the value reported in Figure 3 within the experimental error. It is observed that the work function gradually decreased with sequential ZnPc deposition. For the ZnPc thicknesses of 0.5, 1.0, 2.0, 4.0, and 8.0 nm, the corresponding work functions were 4.94, 4.88, 4.79, 4.73, and 4.67 eV, respectively.

In the HOMO region (Figure 7b), the spectral features associated with ZnPc were comparable to those observed at the PEDOT:PSS/ZnPc interface, and their intensities increased with increasing thickness. The magnified view presented in Figure 7c clearly shows the HOMO onset at 0.26 eV. Importantly, this HOMO onset remains constant at 0.26 eV regardless of the ZnPc thickness, mirroring the behavior observed at the pristine PEDOT:PSS/ZnPc interface. The absence of any notable energy-level shifts is consistent with the XPS results shown in Figure 6.

**Figure 8:**
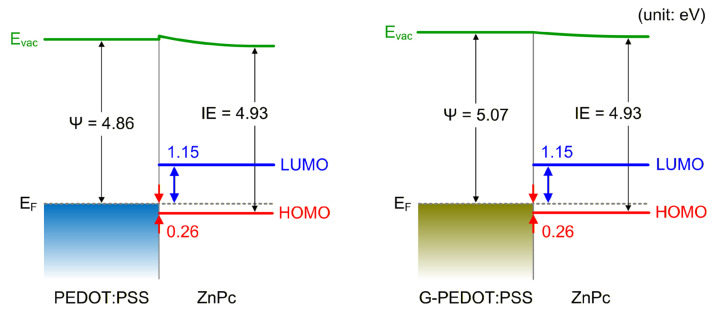
Energy-level diagrams of PEDOT:PSS/ZnPc and G-PEDOT:PSS (10 wt%)/ZnPc.

Figure 8 illustrates the energy-level alignment at the PEDOT:PSS/ZnPc and G-PEDOT:PSS/ZnPc interfaces. The LUMO level of ZnPc was estimated from its transport gap (1.41 eV) [43]. Identical IEs of ZnPc were obtained in both systems (4.93 eV), confirming the sufficient thickness and high reproducibility of ZnPc. For planar organic molecules, both interface dipole formation and variations in the molecular orientation can influence the work function [44,45]. To decouple these effects, a detailed theoretical analysis and precise determination of the molecular orientation are required; however, these aspects are beyond the scope of the present study. Therefore, we report only the overall variation in the work function, without discussing the individual contributions of these two factors. It is worth noting that the vacuum level (E_vac_) and the ZnPc HOMO/LUMO levels are depicted with different curvatures to suggest a variation in IE induced by thickness-dependent changes in molecular orientation; this evolution appears to manifest as the opposing SECO shifts shown in Figure 5a.

The pristine PEDOT:PSS exhibits a work function of 4.86 eV, resulting in the hole and electron injection barriers of 0.26 and 1.15 eV, respectively. This alignment favors hole injection while hindering electron injection. Comparably low hole-injection barriers for ZnPc have also been reported for other substrates with high work functions such as MoO_3_, 1,4,5,8,9,11-hexaazatriphenylene hexacarbonitrile (HAT-CN), and fluorinated self-assembled monolayers (SAMs) [35,46,47]. For substrates with work functions exceeding 4.86 eV, the hole injection barriers remain nearly constant at 0.2–0.3 eV due to Fermi-level pinning, consistent with the experimental uncertainty. The results are summarized in Table 2.

Upon graphene doping, the work function increases to 5.07 eV, corresponding to an increase of 0.21 eV compared to pristine PEDOT:PSS, while the hole and electron injection barriers remain unchanged at 0.26 and 1.15 eV, respectively. This lack of change indicates that the energy level of ZnPc was pinned to the Fermi level of PEDOT: PSS. In other words, the slope parameter, defined as the ratio of the change in the hole injection barrier to the change in the substrate work function, was zero, indicating Fermi-level pinning at both interfaces.

According to the ICT model, when the substrate work function is equal to the hole ICT level (E_ICT+_) of an organic semiconductor, further increases in the substrate work function no longer reduce the hole injection barrier [14]. In this study, the E_ICT+_ of ZnPc is estimated to be 0.26 eV, and the work function of PEDOT:PSS is already sufficiently high to pin this level. Consequently, the hole injection barrier was minimized, and any additional increase in the work function no longer led to a further reduction in the barrier. Therefore, the graphene-induced work function increase did not improve hole injection for ZnPc on PEDOT:PSS.

While the hole injection barrier for ZnPc remained unchanged due to the Fermi-level pinning already established by its low IE, this result serves as a critical verification that G-PEDOT:PSS maintains the fundamental ICT alignment mechanism. The increased work function of 5.07 eV in G-PEDOT:PSS is expected to be more impactful for p-type organic semiconductors with higher IEs (i.e., higher E_ICT+_ values), such as 4,4′-bis (N-carbazolyl)-1,1′-biphenyl (CBP, IE ≈ 6.2 eV), 4,4′,4″-tris(carbazole-9-yl)triphenylamine (TCTA, IE ≈ 5.8 eV), and 1,3-bis (N-carbazolyl) benzene (mCP, IE ≈ 6.0 eV). For such materials, the tuned work function would effectively reduce the hole injection barrier before reaching the pinning regime, thereby broadening the practical utility of graphene doping for a wider range of high-efficiency organic electronic devices.

## 4. Conclusions

The electronic structure of G-PEDOT:PSS was systematically investigated using XPS and UPS measurements. The work function of PEDOT:PSS increased from 4.86 to 5.03 eV with increasing graphene concentrations. To assess the impact of this modification on hole injection, energy-level alignment with ZnPc was examined via in situ analysis. Both the PEDOT:PSS/ZnPc and G-PEDOT:PSS/ZnPc interfaces exhibited identical hole injection barriers of 0.26 eV. This lack of variation indicates that the E_ICT+_ of ZnPc is pinned to the Fermi level of PEDOT:PSS owing to the relatively low IE of ZnPc.

While the energy-level alignments characterized in this study were performed under ultrahigh vacuum (UHV) conditions to ensure intrinsic measurement accuracy, it is important to note that PEDOT:PSS is sensitive to environment factors such as humidity and processing history in practical applications. Ambient exposure or electrical bias during device operation may induce further interfacial modifications. Nonetheless, the fundamental Fermi-level pinning mechanisms and work function trends identified through our UHV analysis provide an essential framework for understanding the underlying charge injection processes in G-PEDOT:PSS-based optoelectronic devices.

Although graphene doping did not reduce the hole injection barrier for ZnPc, the observed work function increase suggests that graphene doping may be beneficial for other p-type organic semiconductors with larger IEs. Accordingly, graphene doping serves as an effective strategy for tuning the work function of PEDOT:PSS, thereby improving the hole-injection efficiency in a wide range of organic and hybrid optoelectronic devices.

## Figures and Tables

**Table 1:** Deconvolution and curve-fitting parameters of the XPS C 1s spectra for PEDOT:PSS films with different graphene doping concentrations. The three primary peak components were deconvoluted using a fixed full-width at half maximum of 1.4 eV and a Gaussian/Lorentzian ratio of 0.4. The π–π* satellite is a shake-up peak due to the loss of photoelectron kinetic energy caused by the excitation of the π (HOMO) to π* (LUMO) transition.

Sample	Component	Binding Energy (eV)	Area (Arb. Unit)	Ratio (%)
PEDOT:PSS	C–C	284.2	28,500	79.1
	C–S	285.2	4500	12.5
	C–O	286.2	2500	6.9
	π–π* satellite	290.7	530	1.5
2 wt%	C–C	284.1	20,500	79.0
	C–S	285.1	3000	11.6
	C–O	286.1	1800	6.9
	π–π* satellite	290.6	664	2.6
5 wt%	C–C	284.1	20,000	78.9
	C–S	285.1	2900	11.4
	C–O	286.1	1780	7.0
	π–π* satellite	290.6	680	2.7
10 wt%	C–C	284.1	19,000	79.5
	C–S	285.1	2700	11.3
	C–O	286.1	1700	7.1
	π–π* satellite	290.6	506	2.1
20 wt%	C–C	284.1	11,900	82.3
	C–S	285.1	1200	8.3
	C–O	286.1	1100	7.6
	π–π* satellite	290.6	257	1.8

**Table 2:** Hole injection barriers of ZnPc on various substrates under Fermi-level pinning conditions.

Substrate	Work Function (eV)	Hole Injection Barrier (eV)
PEDOT:PSS	4.86	0.26
G-PEDOT:PSS	5.07	0.26
HAT-CN [35]	5.35	0.20
F-SAM [46]	5.38	0.3
MoO_3_ [47]	6.6	0.3

## Data Availability

The original contributions presented in this study are included in the article. Further inquiries can be directed to the corresponding author.

## References

[1] Fan X., Stott N.E., Zeng J., Li Y., Ouyang J., Chu L., Song W. (2023). PEDOT:PSS materials for optoelectronics, thermoelectrics, and flexible and stretchable electronics. J. Mater. Chem. A.

[2] Kayser L.V., Lipomi D.J. (2019). Stretchable conductive polymers and composites based on PEDOT and PEDOT:PSS. Adv. Mater..

[3] Hu L., Song J., Yin X., Su Z., Li Z. (2020). Research progress on polymer solar cells Based on PEDOT:PSS electrodes. Polymer.

[4] Xia Y., Dai S. (2021). Review on applications of PEDOTs and PEDOT:PSS in perovskite solar cells. J. Mater. Sci. Mater. Electron..

[5] Fan Z., Ouyang J. (2019). Thermoelectric properties of PEDOT:PSS. Adv. Electron. Mater..

[6] Wen Y., Xu J. (2017). Scientific importance of water-processable PEDOT–PSS and preparation, challenge and new application in sensors of its film electrode: A review. J. Polym. Sci. A Pol. Chem..

[7] Greczynski G., Kugler T., Keil M., Osikowicz W., Fahlman M., Salaneck W.R. (2001). Photoelectron spectroscopy of thin films of PEDOT–PSS conjugated polymer blend: A mini-review and some new results. J. Electron Spectrosc. Relat. Phenom..

[8] Shi H., Liu C., Jiang Q., Xu J. (2015). Effective approaches to improve the electrical conductivity of PEDOT:PSS: A review. Adv. Electron. Mater..

[9] Adekoya G.J., Sadiku R.E., Ray S.S. (2021). Nanocomposites of PEDOT:PSS with graphene and its derivatives for flexible electronic applications: A review. Macromol. Mater. Eng..

[10] Pei S., Xiong X., Zhong W., Xue X., Zhang M., Hao T., Zhang Y., Liu F., Zhu L. (2022). Highly efficient organic solar cells enabled by the incorporation of a sulfonated graphene doped PEDOT:PSS interlayer. ACS Appl. Mater. Interfaces.

[11] Wu T., Shi X.-L., Deng Y.-Y., Liu Y.-M., Zhu M., Liu W.-D., Li M., Yue F., Huang P., Chen Z.-G. (2025). Incorporating graphene quantum dots boosts thermoelectric performance of PEDOT:PSS films. Chem. Eng. J..

[12] Redondo-Obispo C., Ripolles T.S., Cortijo-Campos S., Álvarez A.L., Climent-Pascual E., de Andrés A., Coya C. (2020). Enhanced stability and efficiency in inverted perovskite solar cells through graphene doping of PEDOT:PSS hole transport layer. Mater. Des..

[13] Shin W., Choi S., Moon J., Lee H. (2025). Electronic structure of graphene films prepared from water dispersions and their energy level alignments with organic semiconductors. J. Chem. Phys..

[14] Braun S., Salaneck W.R., Fahlman M. (2009). Energy-level alignment at organic/metal and organic/organic interfaces. Adv. Mater..

[15] Greiner M.T., Helander M.G., Tang W.-M., Wang Z.-B., Qiu J., Lu Z.-H. (2012). Universal energy-level alignment of molecules on metal oxides. Nat. Mater..

[16] Yang J.-P., Shang L.-T., Bussolotti F., Cheng L.-W., Wang W.-Q., Zeng X.-H., Kera S., Li Y.-Q., Tang J.-X., Ueno N. (2017). Fermi-level pinning appears upon weak electrode-organic contact without gap states: A universal phenomenon. Org. Electron..

[17] Yoo J., Jung K., Jeong J., Hyun G., Lee H., Yi Y. (2017). Energy level alignment at C_60_/DTDCTB/PEDOT:PSS interfaces in organic photovoltaics. Appl. Surf. Sci..

[18] Hwang J., Amy F., Kahn A. (2006). Spectroscopic study on sputtered PEDOT PSS: Role of surface PSS layer. Org. Electron..

[19] Shin D., Kang D., Lee J.-B., Ahn J.-H., Cho I.-W., Ryu M.-Y., Cho S.W., Jung N.E., Lee H., Yi Y. (2019). Electronic structure of nonionic surfactant-modified PEDOT:PSS and its application in perovskite solar cells with reduced interface recombination. ACS Appl. Mater. Interfaces.

[20] Wang R., Wang Y., Wu C., Zhai T., Yang J., Sun B., Duhm S., Koch N. (2020). Direct observation of conductive polymer induced inversion layer in n-Si and correlation to solar cell performance. Adv. Funct. Mater..

[21] Jung S., Choi S., Shin W., Oh H., Oh J., Ryu M.-Y., Kim W., Park S., Lee H. (2023). Enhancement in power conversion efficiency of perovskite solar cells by reduced non-radiative recombination using a Brij C10-mixed PEDOT:PSS hole transport layer. Polymers.

[22] Zhang Y., Wang Q., Hu F., Wang Y., Wu D., Wang R., Duhm S. (2023). Photoelectron spectroscopy reveals the impact of solvent additives on poly(3,4-ethylenedioxythiophene):poly(styrenesulfonate) thin film formation. ACS Phys. Chem. Au.

[23] Unsworth N.K., Hancox I., Argent Dearden C., Sullivan P., Walker M., Lilley R.S., Sharp J., Jones T.S. (2014). Comparison of dimethyl sulfoxide treated highly conductive poly(3,4-ethylenedioxythiophene):poly(styrenesulfonate) electrodes for use in indium tin oxide-free organic electronic photovoltaic devices. Org. Electron..

[24] Choi S., Kim W., Shin W., Han H.J., Park C., Oh H., Jung S., Park S., Lee H. (2023). Electronic structure modification of polymeric PEDOT:PSS electrodes using the nonionic surfactant Brij C10 additive for significant sheet resistance reduction. Appl. Surf. Sci..

[25] Mahato S., Puigdollers J., Voz C., Mukhopadhyay M., Mukherjee M., Hazra S. (2020). Near 5% DMSO is the best: A structural investigation of PEDOT: PSS thin films with strong emphasis on surface and interface for hybrid solar cell. Appl. Surf. Sci..

[26] Xie Y., Xuan X., Tang Y., Bi Z., Wang P., Zou J., Kang Y., Zhang A., Yamauchi Y., Yang C. (2025). Synergistic Mo/V-implanted 2D M_3_X_2_ MXene nanoarchitectures for enhanced structural stability and ultrahigh proton storage performance. Adv. Energy Mater..

[27] Xuan X., Xie Y., Tang Y., Zhou J., Bi Z., Zou J., Lei Y.-a., Gao J., Li L., Zhang A. (2025). Revealing the ionic storage mechanisms of Mo_2_VC_2_T_z_ (MXene) in multiple aqueous electrolytes for high-performance supercapacitors. Chem. Eng. J..

[28] Zhou J., Chang N., Tang Y., Xie Y., Xuan X., Bi Z., Zou J., Li L., Liu M., Yang C. (2025). Uncovering the strengthening mechanisms of metal vacancies in the structure and capacitance performance of defect-controlled Mo_2−□_CT_z_ MXene. Chem. Eng. J..

[29] Musso T., Kumar P.V., Foster A.S., Grossman J.C. (2014). Graphene oxide as a promising hole injection layer for MoS_2_-based electronic devices. ACS Nano.

[30] Sygellou L., Paterakis G., Galiotis C., Tasis D. (2016). Work function tuning of reduced graphene oxide thin films. J. Phys. Chem. C.

[31] Jia S., Sun H.D., Du J.H., Zhang Z.K., Zhang D.D., Ma L.P., Chen J.S., Ma D.G., Cheng H.M., Ren W.C. (2016). Graphene oxide/graphene vertical heterostructure electrodes for highly efficient and flexible organic light emitting diodes. Nanoscale.

[32] Shin W., Lee H. (2025). Improved hole injection at the zinc phthalocyanine/solution-processed graphene interface via ultraviolet–ozone treatment. Appl. Sci. Converg. Technol..

[33] Yun D.-J., Ra H., Kim J., Hwang I., Lee J., Rhee S.-W., Chung J. (2012). Characterizing annealing effect of poly (3,4-ethylenedioxythiophene) polymerized with poly (4-styrenesulfonate) conjugated film on the molecular arrangement and work function by core-level and valence-level band spectra. ECS J. Solid State Sci. Technol..

[34] Luo Z., Shang J., Lim S., Li D., Xiong Q., Shen Z., Lin J., Yu T. (2010). Modulating the electronic structures of graphene by controllable hydrogenation. Appl. Phys. Lett..

[35] Joo E., Hur J.W., Ko J.Y., Kim T.G., Hwang J.Y., Smith K.E., Lee H., Cho S.W. (2023). Effects of HAT-CN layer thickness on molecular orientation and energy-level alignment with ZnPc. Molecules.

[36] Xu B., Sai-Anand S., Jeong H.-M., Kim S.-W., Kim J.-S., Kwon J.-B., Kang S.-W. (2018). Improving air-stability and performance of bulk heterojunction polymer solar cells using solvent engineered hole selective interlayer. Materials.

[37] Zhao Y., Su H., Liu Q., Zhang L., Lv M., Cheng P., Liu D., He D. (2020). Improvement of the optoelectrical properties of a transparent conductive polymer via a simple mechanical pressure treatment. ACS Omega.

[38] Kera S., Yabuuchi Y., Yamane H., Setoyama H., Okudaira K.K., Kahn A., Ueno N. (2004). Impact of an interface dipole layer on molecular level alignment at an organic-conductor interface studied by ultraviolet photoemission spectroscopy. Phys. Rev. B.

[39] Wang Q.-K., Wang R.-B., Shen P.-F., Li C., Li Y.-Q., Liu L.-J., Duhm S., Tang J.-X. (2015). Energy level offsets at lead halide perovskite/organic hybrid interfaces and their impacts on charge separation. Adv. Mater. Interfaces.

[40] Jung K., Park S., Yoo J., Jung N.E., Moon B.J., Lee S.H., Yi Y., Lee H. (2022). Elucidation of hole transport mechanism in efficient energy cascade organic photovoltaics using triple donor system. Appl. Surf. Sci..

[41] Whitten J.E. (2023). Ultraviolet photoelectron spectroscopy: Practical aspects and best practices. Appl. Surf. Sci. Adv..

[42] Zhu L., Xu X. (2025). Surface charging on insulating films with different thicknesses in UPS. Appl. Sci..

[43] Tietze M.L., Tress W., Pfützner S., Schünemann C., Burtone L., Riede M., Leo K., Vandewal K., Olthof S., Schulz P. (2013). Correlation of open-circuit voltage and energy levels in zinc-phthalocyanine: C_60_ bulk heterojunction solar cells with varied mixing ratio. Phys. Rev. B.

[44] Chen W., Huang H., Chen S., Huang Y.L., Gao X.Y., Wee A.T.S. (2008). Molecular orientation-dependent ionization potential of organic thin films. Chem. Mater..

[45] Jeong J., Park S., Kang S.J., Lee H., Yi Y. (2016). Impacts of molecular orientation on the hole injection barrier reduction: CuPc/HAT-CN/Graphene. J. Phys. Chem. C.

[46] Akaike K., Koch N., Heimel G., Oehzelt M. (2015). The impact of disorder on the energy level alignment at molecular donor–acceptor interfaces. Adv. Mater. Interfaces.

[47] Wang H., Yang X., Dou W., Wang P., Ye Q., Yang X., Li B., Mao H. (2019). Impact of graphene work function on the electronic structures at the interface between graphene and organic molecules. Nanomaterials.

